# Normal Appearing Ischaemic Brain Tissue on CT and Outcome After Intravenous Alteplase

**DOI:** 10.3389/fradi.2022.902165

**Published:** 2022-06-22

**Authors:** Grant Mair, Joanna M. Wardlaw

**Affiliations:** ^1^Centre for Clinical Brain Sciences, University of Edinburgh, Edinburgh, United Kingdom; ^2^UK Dementia Research Institute Centre at the University of Edinburgh, Edinburgh, United Kingdom

**Keywords:** CT, ischaemic, stroke, alteplase, functional outcome

## Abstract

**Background and Aims:**

The visibility of ischaemic brain lesions on non-enhanced CT increases with time. Obviously hypoattenuating lesions likely represent infarction. Conversely, viable ischaemic brain lesions may be non-visible on CT. We tested whether patients with normal appearing ischaemic brain tissue (NAIBT) on their initial CT are identifiable, and if NAIBT yields better outcomes with alteplase.

**Methods:**

With data from the Third International Stroke Trial (IST-3, a large randomized-controlled trial of intravenous alteplase for ischaemic stroke) we used receiver-operating characteristic analysis to find a baseline National Institutes of Health Stroke Scale (NIHSS) threshold for identifying patients who developed medium-large ischaemic lesions within 48 h. From patients with baseline CT (acquired <6 h from stroke onset), we used this NIHSS threshold for selection and tested whether favorable outcome after alteplase (6-month Oxford Handicap Score 0–2) differed between patients with NAIBT vs. with those with visible lesions on baseline CT using binary logistic regression (controlled for age, NIHSS, time from stroke onset to CT).

**Results:**

From 2,961 patients (median age 81 years, median 2.6 h from stroke onset, 1,534 [51.8%] female, 1,484 [50.1%] allocated alteplase), NIHSS>11 best identified those with medium-large ischaemic lesions (area under curve = 0.79, sensitivity = 72.3%, specificity = 71.9%). In IST-3, 1,404/2,961 (47.4%) patients had baseline CT and NIHSS>11. Of these, 745/1,404 (53.1%) had visible baseline ischaemic lesions, 659/1,404 (46.9%) did not (NAIBT). Adjusted odds ratio for favorable outcome after alteplase was 1.54 (95% confidence interval, 1.01–2.36), p = 0.045 among patients with NAIBT vs. 1.61 (0.97–2.67), *p* = 0.066 for patients with visible lesions, with no evidence of an alteplase-NAIBT interaction (*p*-value = 0.895).

**Conclusions:**

Patients with ischaemic stroke and NIHSS >11 commonly develop sizeable ischaemic brain lesions by 48 h that may not be visible within 6 h of stroke onset. Invisible ischaemic lesions may indicate tissue viability. In IST-3, patients with this clinical-radiological mismatch allocated to alteplase achieved more favorable outcome than those allocated to control.

## Introduction

In the hours following the onset of an ischaemic stroke, the visibility of ischaemic brain lesions on non-enhanced CT changes (1). As the brain injury progresses, there is greater tissue oedema which appears darker (i.e., is more hypoattenuating) and swollen compared to normal brain on CT (2). The more visible an ischaemic brain lesion appears on CT, the more likely it is irreversibly injured or infarcted (and the easier it is to find). Conversely, ischaemic brain tissue that remains viable is more likely to be non-visible or only subtly visible (and more likely to be overlooked) on CT, i.e., there is a clinical-radiological mismatch (3). This process is imperfectly associated with the time elapsed after stroke symptom onset since patients may or may not have collateral arterial blood supply to the affected brain (4). The presence of collaterals can sustain injured tissue for extended time periods during which reperfusion therapy is likely to be most effective, whereas patients without collaterals are likely to progress more quickly to infarct and thus may benefit less from reperfusion therapy.

Intravenous alteplase is an effective treatment for acute ischaemic stroke but treatment benefit reduces with time (5). Therefore, alteplase is mostly restricted to patients where stroke onset time is known and treatment can be initiated within set time limits; 4.5 h after onset in Europe, within 3 h of onset in the USA (6, 7). Recent randomized-controlled trials have shown that when stroke onset time is not known or there is a delay to presentation up to 9 h, more advanced imaging methods including CT perfusion and MRI may be used to identify patients with persistent ischaemic but viable brain tissue who can still safely benefit from intravenous alteplase (8, 9). Many stroke centers will therefore now offer CT angiography and/or CT perfusion (or the MRI equivalents) to directly image arterial collaterals and/or the associated bypass brain perfusion for patients where the time of ischaemic stroke onset is uncertain or beyond conventional treatment time limits. However, these imaging methods are not always available, especially outside major treatment centers in advanced healthcare systems (10, 11). Therefore, we sought to investigate whether similar inferences of brain tissue viability (and thus, treatment eligibility) can be obtained using clinical examination and simple, routinely-acquired non-enhanced CT brain imaging alone. From among patients with acute symptoms of stroke but no visible brain lesion on non-enhanced CT, if we can differentiate those with truly non-visible (or not yet visible) lesions from those without lesions (e.g., alternative stroke mimic diagnosis), or with lesions that will rarely ever be visible on baseline CT (e.g., lacunar or tiny cortical infarct), we might identify a cohort of patients with injured but still viable normal-appearing ischaemic brain tissue (NAIBT). In other words, we anticipate that by identifying the clinical-radiological mismatch described above, non-enhanced CT might be used to identify patients that can be safely treated with intravenous alteplase when stroke onset time is unknown, or when the patient presents later than conventional treatment time limits, and when advanced imaging methods are not available.

We aimed to test if it is feasible to identify patients with NAIBT using only standard baseline clinical data in stroke including non-enhanced CT, and to determine whether the intravenous alteplase effect on outcome is modified by NAIBT.

## Materials and Methods

### Study Design

We used data from a large randomized-controlled trial that tested intravenous alteplase after ischaemic stroke to:

1. Identify the baseline clinical features of patients who develop medium-large ischaemic brain lesions following ischaemic stroke.
2. Use these baseline clinical features to best define a trial subgroup most likely to have ischaemic brain lesions.
3. Separate subgroup patients into those with and without *visible* ischaemic brain lesions on baseline non-enhanced CT (i.e., with and without NAIBT).
4. Compare the alteplase effect on outcome among patients with and without NAIBT.

### Data

The Third International Stroke Trial (IST-3) recruited 3,035 patients with ischaemic stroke under 6 h from symptom onset (12). IST-3 recruited patients from 156 hospitals in 12 countries over 12 years (2000–2012). Patients were randomized to receive intravenous alteplase (0.9 mg/kg) or open control. All patients in IST-3 were assessed locally by experienced stroke clinicians at baseline for stroke severity using the National Institutes of Health Stroke Scale (NIHSS), and stroke syndrome using the Oxfordshire Community Stroke Project (OCSP) classification (13). Routine brain imaging (mostly CT, but MRI was accepted) was acquired based on local protocols at baseline for all patients, and again at 24–48 h after stroke onset for those that survived. To maximize trial recruitment but ensure adequate image quality, participating sites had to meet minimum image acquisition standards (e.g., maximum slice thickness 10 mm with no slice gap), but strict or specific image parameters were not otherwise imposed (14). Brain imaging was collected centrally for review by a panel of ten masked experts (8 neuroradiologists and 2 stroke neurologists, all experienced in acute stroke imaging interpretation and tested to have >0.7 kappa agreement for acute lesion detection) (15) using a standardized and clinically-validated online review platform (Systematic Image Review System, SIRS: https://sirs2.ccbs.ed.ac.uk/sirs2) (14). Symptomatic intracerebral hemorrhage (SICH) was defined as any clinically-important worsening of deficit measured on a valid stroke scale or recurrent stroke symptoms associated with significant intracranial hemorrhage within seven days of stroke onset (14). Functional outcome was assessed at 6 months after stroke onset using the Oxford Handicap Scale (OHS). OHS is a seven-point scalar measure from 0 (normal), through increasingly severe grades of disability (1–5), to 6 (death). IST-3 was registered (ISRCTN25765518) and had ethical approval (MREC/99/0/78). Consent was obtained for all patients.

### Imaging Assessment

Experts assessed imaging masked to all other clinical data (including other imaging) for a range of acute brain changes relating to stroke including the presence, location, and extent of visible ischaemic brain lesions (with attenuation changes and swelling scored separately), hyperattenuating arteries (an imaging surrogate highly specific for arterial obstruction), and hemorrhage. Ischaemic lesion extent was assessed using both the ASPECTS (Alberta Stroke Program Early CT Score) and IST-3 methods (15, 16). ASPECTS can be used to assess lesion extent only within the middle cerebral artery territory on an 11-point ordinal scale from normal (10) to involvement of the entire MCA territory (0). The IST-3 method allows scoring of the whole brain on a 5-point ordinal score from normal (0) to very large (4). We have previously tested reader-reliability for seven of the 10 expert imaging panel members in IST-3. Krippendorff's Alpha results (0 = no agreement, 1 = perfect agreement) were as follows. Inter-rater reliability was 0.66 for the detection of acute ischaemic brain changes (substantial agreement), 0.56 for ASPECTS, 0.59 for the IST-3 lesion extent score (both moderate agreement), 0.37 for identifying hyperattenuating arteries (fair agreement) (17).

For the purposes of this analysis, we defined medium-large ischaemic brain lesions as ASPECTS 0–7 or IST-3 method score 2–4 (i.e., excluding small cortical and lacunar lesions). If these scores were discrepant, we based classification primarily on the IST-3 score due to its assessment of the whole brain. Patients were considered to have medium-large ischaemic lesions if visible on the assessment of either baseline or follow-up imaging (lesions did not need to be visible on both).

### Patient Selection

For the current analysis, we only include patients who had non-enhanced CT brain imaging acquired at baseline. We did not exclude patients based on the presence or modality of any 24–48 h follow-up imaging. However, patients without follow-up imaging were only used to define the subgroup when a baseline lesion was identified, those without visible lesions on baseline imaging were not used to define the subgroup since lesions may not be visible at baseline.

### Data Analysis and Statistics

#### Identify Baseline Clinical Features of Patients Who Develop Medium-Large Lesions

For univariate comparisons of groups with and without medium-large ischaemic lesions, we used *t-*tests, chi-square tests, and Mann Whitney-U tests, as appropriate. We applied a Bonferroni adjustment to univariate comparisons to avoid Type 1 errors.

#### Define Trial Subgroup Most Likely to Have Medium-Large Lesions

We used receiver-operating characteristic (ROC) analysis to find a baseline NIHSS threshold for identifying patients who had or would develop medium-large ischaemic brain lesions at any imaging time point across the whole group and in sensitivity analyses for groups where medium-large lesions were more likely according to univariate analyses: (1) excluding patients with a lacunar stroke syndrome, and (2) only including patients with a hyperattenuating artery. We selected the ROC result with the greatest area under the curve (AUC) for subgroup analysis.

#### Compare the Alteplase Effect on Outcome Among Subgroup Patients With and Without NAIBT

We then tested whether SICH rates and favorable functional outcome after alteplase (six-month OHS 0–2) differed between subgroup patients with NAIBT vs. with those with visible lesions on baseline CT using binary logistic regression (controlled for age, NIHSS, time from stroke onset to CT).

We used SPSS version 25, IBM Corporation (Armonk, USA) for all analyses. We preferentially report 95% confidence intervals (95%CI) but include *p*-values (<0.05 considered significant, unless adjusted) where appropriate.

## Results

Of 3,035 patients recruited in IST-3, 2,961 had centrally-reviewed non-enhanced brain CT acquired at baseline: 1,534 (51.8%) female, median age 81 years (inter-quartile range, IQR 72–86 years), median NIHSS 11 (IQR 6–18), 323 (10.9%) had a lacunar stroke syndrome, median 2.6 h (IQR 1.8–3.6 h) from stroke symptom onset to baseline CT, 716 (24.2%) had a hyperattenuating artery on baseline CT, 1,421/2,890 (49.2% of those available to define subgroup) had a medium-large ischaemic lesion identified on either baseline or 24–48 h follow-up imaging, 1,484 (50.1%) were treated with alteplase, 116 (3.9%) suffered SICH, and 1,053 (35.6%) had an OHS 0–2 while 797 (26.9%) were dead at 6 months.

### Baseline Clinical Features of Patients Who Develop Medium-Large Ischaemic Lesions

**Figure 1:**
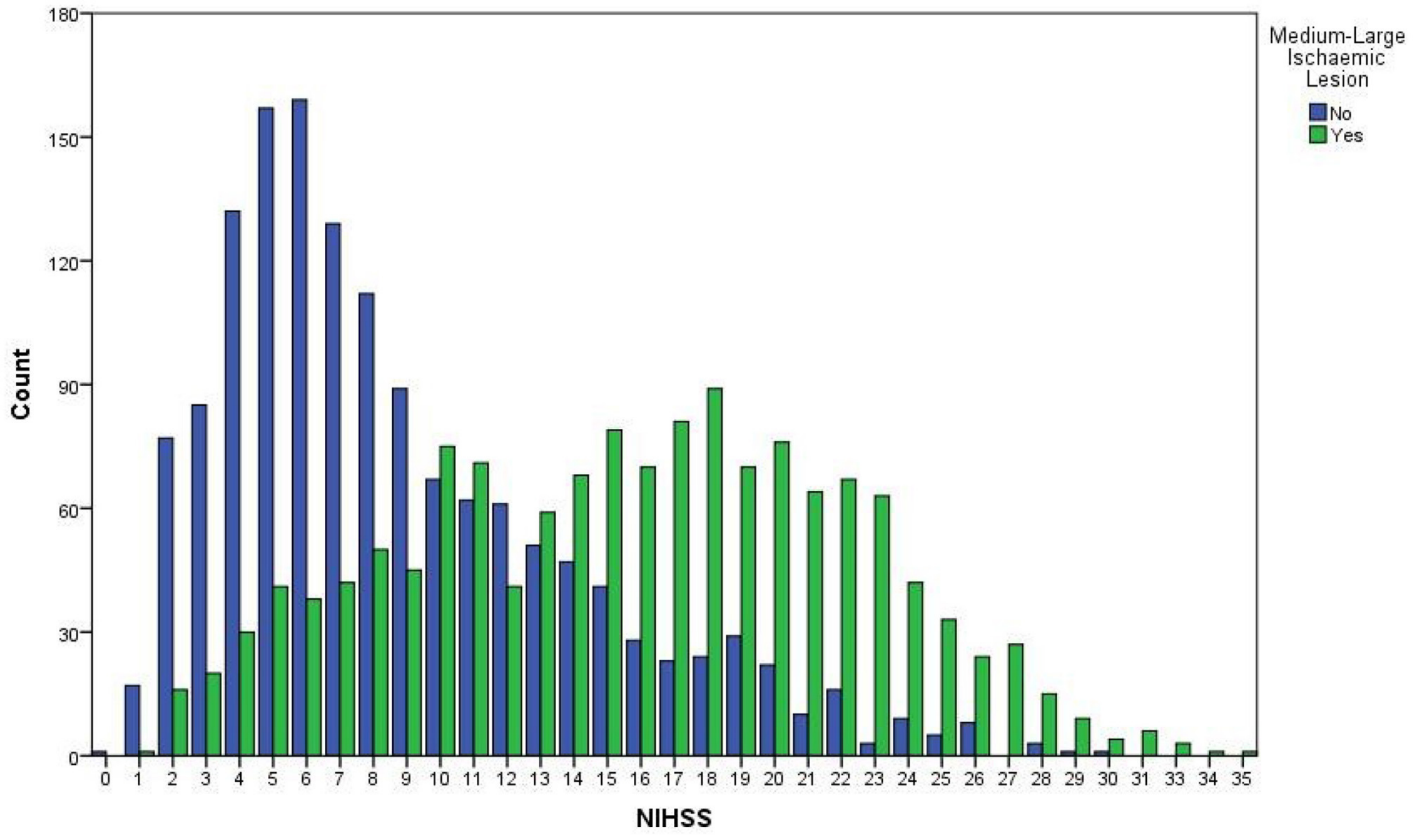
Overlapping histograms for baseline NIHSS among patients with and without a medium-large ischaemic lesion, *n* = 2,890. NIHSS, National Institutes of Health Stroke Scale.

Table 1 compares the baseline clinical and demographic features of 2,890 patients with and without a medium-large ischaemic lesion identified at any time point in IST-3 (i.e., excluding 71 patients without follow-up imaging and no visible ischaemic lesion on baseline imaging). Patients with medium-large lesions were significantly more likely to be older, to have a higher baseline NIHSS (Figure 1), and were more likely to have a visible hyperattenuating artery at baseline. The clinical stroke syndrome defined at baseline was distributed differently between groups with (total anterior circulation strokes more common) and without (lacunar, posterior circulation, and partial anterior circulation strokes more common) medium-large lesions at any time point.

**Table 1:** Baseline clinical and demographic differences between those with and without a confirmed medium-large ischaemic lesion at any time, *n* = 2,890.

**Variable**	**Medium-large lesion**		***p*** **-value**
	**Yes (1,421)**	**No (1,469)**	
Age, years	82 (73–86)	81 (71–85)	**0.001**
Sex, female	769 (54.1%)	726 (49.4%)	0.012
Hyperattenuating artery on baseline CT	566 (39.8%)	145 (9.9%)	**<0.001**
NIHSS	16 (10–21)	7 (5–12)	**<0.001**
NIHSS range	1–35	0–30	
Stroke syndrome (2,886)			
LACS	45	272	
PACS	402	680	**<0.001**
POCS	63	168	
TACS	909	347	

*Results are n (%) or median (inter-quartile range). NIHSS, National Institutes of Health Stroke Scale; LACS, Lacunar Stroke; PACS, Partial Anterior Circulation Stroke; POCS, Posterior Circulation Stroke; TACS, Total Anterior Circulation Stroke. P-values in bold are significant following Bonferroni correction for multiple testing*.

### Subgroup Most Likely to Have Medium-Large Ischaemic Lesions

**Figure 2:**
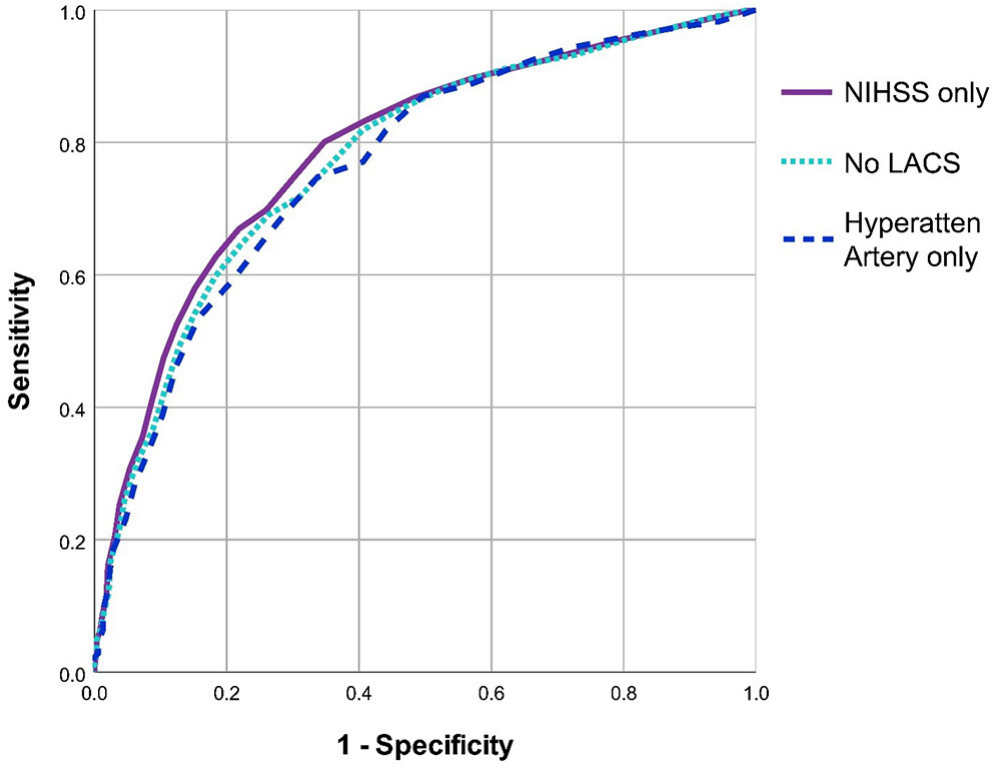
Comparison of ROC curves for three methods used to define the subgroup most likely to have medium-large ischaemic lesions. All three ROC analyses sought an NIHSS threshold to discriminate patients with and without medium-large ischaemic lesions. The first analysis includes all patients (i.e., uses NIHSS alone, *n* = 2890), the second excludes patients with a lacunar stroke syndrome (*n* = 2569), the third only includes patients with a hyperattenuating artery on baseline brain CT (*n* = 711). NIHSS, National Institutes of Health Stroke Scale. LACS, Lacunar Stroke.

On ROC analysis, baseline NIHSS >11 was the best discriminator of true medium-large ischaemic lesion presence in the full cohort (*n* = 2890, AUC = 0.79, 95%CI 0.77–0.81) with sensitivity = 72.3%, specificity = 71.9%, Figure 1. On sensitivity analysis when patients with LACS (and those with no stroke syndrome recorded) were excluded, baseline NIHSS >11 remained the best discriminator but was slightly less effective overall (*n* = 2569, AUC = 0.77, 95%CI 0.76–0.79), sensitivity = 70.4%, specificity = 71.2%. Similarly, when only patients with a hyperattenuating artery on baseline non-enhanced CT were included, baseline NIHSS >13 was the best discriminator but was again less effective and was considerably less well powered than the full cohort for correctly identifying patients with medium-large ischaemic lesions (*n* = 711, AUC = 0.77, 95%CI 0.72–0.81), sensitivity = 70.1%, specificity = 70.3%. Figure 2 compares the three ROC curves.

### Selected Subgroup Patients and Ischaemic Brain Lesion Visibility on Baseline Non-Enhanced CT

1,404 of 2,961 (47.4%) patients in IST-3 had NIHSS >11 at baseline. Of these, 745 (53.1%) had a visible ischaemic lesion at baseline while 659 (46.9%) did not.

### Alteplase Effect on Functional Outcome for Subgroup Patients With and Without NAIBT

**Figure 3:**
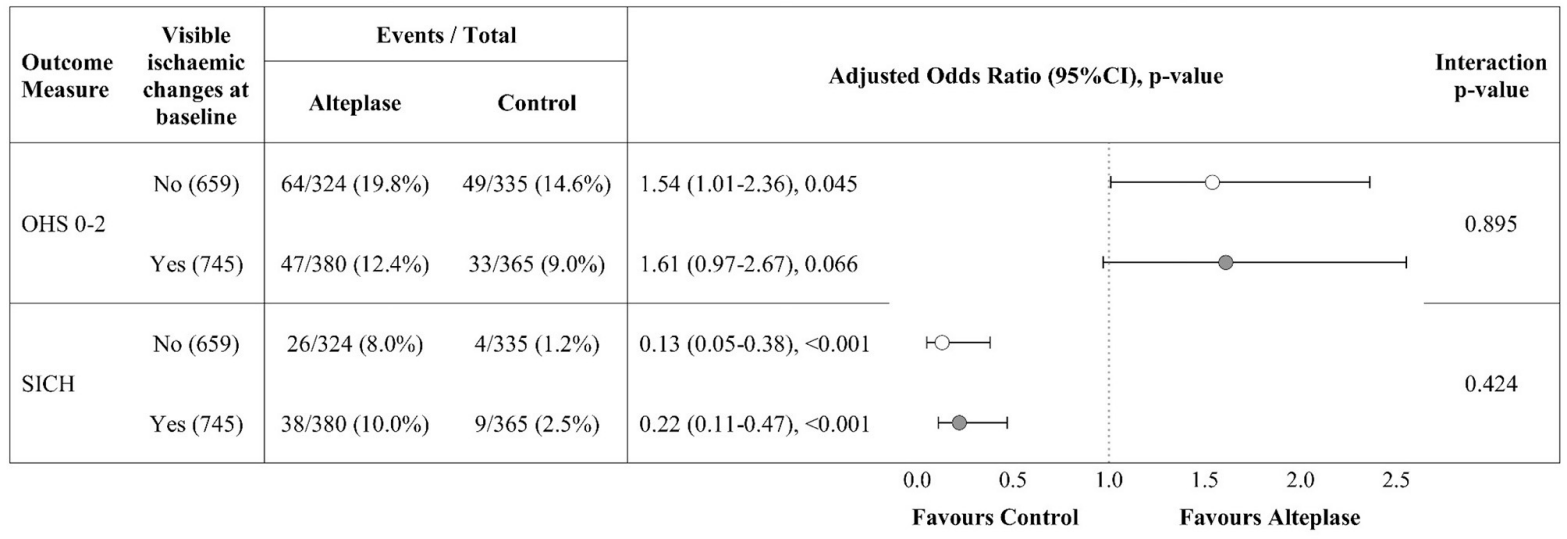
Alteplase effect on outcome for subgroup patients (NIHSS >11) with and without visible ischaemic brain changes on baseline CT, *n* = 1,404. Odds ratios are adjusted for age, NIHSS, time between stroke onset and CT scan. Open circles represent patients with NAIBT, closed circles without NAIBT. NIHSS, National Institutes of Health Stroke Scale; OHS, Oxford Handicap Scale at 6 months; SICH, Symptomatic Intracerebral Hemorrhage; NAIBT, Normal appearing ischaemic brain tissue.

Figure 3 compares the results of binary logistic regression for subgroup patients (*n* = 1,404) with and without visible ischaemic lesions on baseline non-enhanced CT (i.e., with and without NAIBT). Patients with NAIBT were significantly more likely to have a good functional outcome after treatment with intravenous alteplase compared with those allocated to the control group (adjusted odds ratio, adjOR = 1.54, 95%CI 1.01–2.36, *p* = 0.045). There was a non-significant trend toward improved outcome after alteplase for patients without NAIBT (adjOR = 1.61, 95%CI 0.97–2.67, p = 0.066). SICH did not differ between patients with visible lesions (47/745, 6.3%) vs. NAIBT (30/659, 4.6%), *p* = 0.163. We did not identify a difference in the alteplase effect on outcome (*p* = 0.895) or rate of SICH (*p* = 0.424) for patients with vs. without NAIBT, i.e., we did not identify any treatment interaction.

## Discussion

By identifying a mismatch between moderate-severe clinical stroke severity and normal CT brain imaging, we have tried to develop a simple method using only routine clinical assessment and non-enhanced CT brain imaging commonly available at baseline to provide a possible surrogate for persistent viability of ischaemic brain tissue up to 6 hours from stroke symptom onset. Using subgroup data from a well-defined, large randomized-controlled trial of intravenous alteplase, we found that patients with NIHSS >11 at baseline were highly likely to develop medium to large ischaemic brain lesions within 48 h of stroke onset. Therefore, patients with NIHSS >11 but no visible ischaemic brain lesion at baseline were considered likely to have “normal appearing ischaemic brain tissue” or NAIBT. We selected patients with medium-large ischaemic lesions for this analysis to increase the likelihood of identifying genuine NAIBT since small lesions are more likely to be overlooked even when truly visible. We then compared the alteplase effect on functional outcome after stroke for patients with and without NAIBT on baseline brain CT. Patients with NAIBT given alteplase were significantly more likely than those allocated to control to have a favorable outcome (no disability or only minimal disability that does not restrict functional independence) 6 months after stroke. Whilst patients without NAIBT (i.e., those with a visible ischaemic lesion on baseline CT acquired before alteplase was given) did not quite reach the threshold for a conventional significant benefit from alteplase in our analysis; however, these subgroups are likely underpowered and we found no significant difference in the effect of alteplase between groups with and without NAIBT, i.e., no evidence of an interaction between alteplase and presence/absence of visible ischaemic tissue.

Previous feasibility studies have looked to simplify patient selection for alteplase when stroke onset time is unknown. The SAIL ON trial assessed the safety of giving intravenous alteplase to patients with wake up stroke (i.e., when time of onset is unknown) where less than one third of the MCA territory was affected by visible ischaemic changes on non-enhanced CT, NIHSS was ≥4, and alteplase could be given within 4.5 h of awakening (18). SAIL ON recruited 20 patients from two centers, all were given intravenous alteplase. No patients in SAIL ON suffered SICH after alteplase, but two had asymptomatic brain hemorrhage. Similarly, TRUST CT was an international multicentre registry-based study of patients with wake-up stroke where ASPECTS was ≥7 on non-enhanced baseline CT alone (patients with advanced imaging were excluded), NIHSS was ≥4, and alteplase could be given within 4.5 h of awakening. Included patients were compared with matched controls not given thrombolysis (19). TRUST CT included 117 patients treated with alteplase and 112 controls: 4 patients given thrombolysis and 1 control suffered SICH but this difference was not significant; patients given alteplase were more likely to see a short term improvement in NIHSS (reduction by 4 points or more at 24 h), with 67 (57%) given alteplase improving vs. 25 (22%) controls, *p* < 0.001; at 90 days patients given alteplase had a non-significant improvement in functional outcome, modified Rankin Score (mRS) 0–1 in 39 (33%) vs. 23 (21%) controls. Both SAIL ON and TRUST CT, therefore, imply that selection for intravenous alteplase among patients with an unknown time of symptom onset is feasible and safe using non-enhanced CT selection alone. Interestingly, both studies are likely to include a mixture of NAIBT and non-NAIBT patients. Given the non-significant lower rate of SICH we demonstrated in those with vs. without NAIBT in our analysis, it is possible our approach might yield better results, but this requires prospective testing.

### Strengths and Limitations

We present data from a large randomized-controlled trial. We have relatively large patient numbers compared to recent similar prospective studies and only randomized trial data can be used to truly understand the impact of imaging biomarkers or other variables on the effect of treatments.

However, as a subgroup pos*t-hoc* analysis, our study is likely underpowered, and IST-3 was not specifically designed to answer the question raised in our exploratory analysis. NIHSS >11 is quite a restrictive threshold which will limit the number of patients our method might be applied to. However, pre-hospital methods designed to rapidly identify patients suitable for thrombectomy with large vessel occlusion define NIHSS thresholds in the range 6–15 (20, 21). Further analyses in different datasets may identify different thresholds. We plan to bolster the current analysis using pooled data from up to 9 randomized-controlled trials of intravenous alteplase (5). Ultimately, our method requires prospective testing, but these exploratory analyses can inform the design of future studies.

In this analysis, we have not tested the assertion that NAIBT on non-enhanced CT is comparable with viable ischaemic tissue demonstrated by other means (3), i.e., with concurrent CT perfusion imaging or MRI, but this work is ongoing (22). Similarly, it was not possible to confirm with IST-3 data that patients with NIHSS >11 and no visible lesion on baseline CT truly had a medium-large non-visible ischaemic lesion (as opposed to a lacunar or small cortical infarct or even a stroke mimic) but our methodology was specifically designed to avoid including small lesions while IST-3 was careful to include only patients with ischaemic stroke. Confirmation that the NIHSS threshold we identified here corresponds to the presence of medium-large ischaemic lesions would require a dataset with concurrent CT perfusion for all patients. Finally, it is well recognized that early ischaemic lesions are difficult to detect on non-enhanced CT (particularly compared to perfusion CT or diffusion weighted MRI) (23), thus if our method was used in clinical practice, there is likely to be some crossover between patients with NAIBT and those where visible medium-large ischaemic lesions are overlooked. Again, the impact of this limitation will be best assessed in a prospective analysis including front-line stroke clinicians.

## Conclusions

In patients with moderate-severe ischaemic stroke but a normal CT brain scan, we postulate that non-visible ischaemic brain injury reflects viable brain tissue and show that in patients with this clinical-radiological mismatch, treatment with intravenous alteplase is likely to improve outcome up to 6 h from symptom onset. Our method requires prospective testing to ascertain whether it can be safely and effectively used to offer treatment to patients where the time of stroke onset is unknown or outside conventional treatment time limits.

## Data Availability Statement

The datasets presented in this study can be found in online repositories. The names of the repository/repositories and accession number(s) can be found below: Edinburgh University DataShare: http://datashare.is.ed.ac.uk/handle/10283/1931.

## Ethics Statement

The studies involving human participants were reviewed and approved by Multi-Centre Research Ethics Committee, Scotland. Ref: MREC/99/0/78. Patients or their representatives provided written informed consent to participate in this study.

## Author Contributions

GM designed the current work, analysed and interpreted the data, and drafted the manuscript. JW helped design IST-3, designed and supervised the IST-3 imaging data collection, co-ordinated the image reading panel, managed all IST-3 imaging analysis, and critically revised the manuscript. All authors contributed to the article and approved the submitted version.

## Funding

IST-3 was funded from many sources but chiefly the UK Medical Research Council (MRC G0400069 and EME 09-800-15) and the UK Stroke Association. GM is the Stroke Association Edith Murphy Foundation Senior Clinical Lecturer (SA L-SMP 18\1000). JW is supported by the UK Dementia Research Institute which receives its funding from DRI Ltd., funded by the UK Medical Research Council, Alzheimer's Society and Alzheimer's Research UK.

## Conflict of Interest

The authors declare that the research was conducted in the absence of any commercial or financial relationships that could be construed as a potential conflict of interest.

## Publisher's Note

All claims expressed in this article are solely those of the authors and do not necessarily represent those of their affiliated organizations, or those of the publisher, the editors and the reviewers. Any product that may be evaluated in this article, or claim that may be made by its manufacturer, is not guaranteed or endorsed by the publisher.
